# Electrochemical Characterization and Inhibiting Mechanism on Calcium Leaching of Graphene Oxide Reinforced Cement Composites

**DOI:** 10.3390/nano9020288

**Published:** 2019-02-19

**Authors:** Wu-Jian Long, Tao-Hua Ye, Li-Xiao Li, Gan-Lin Feng

**Affiliations:** Guangdong Provincial Key Laboratory of Durability for Marine Civil Engineering, Shenzhen Durability Center for Civil Engineering, College of Civil Engineering, Shenzhen University, Shenzhen 518060, China; longwj@szu.edu.cn (W.-J.L.); 2172332301@email.szu.edu.cn (T.-H.Y.); lilixiao@szu.edu.cn (L.-X.L.)

**Keywords:** graphene oxide, cement composites, calcium leaching, electrochemical impedance spectroscopy, scanning electron microscope, inhibiting mechanism

## Abstract

Calcium leaching is a degradation progress inside hardened cement composites, where Ca^2+^ ions in cement pore solution can migrate into the aggressive solution. In this work, calcium leaching of graphene oxide (GO) reinforced cement composites was effectively characterized by combined techniques of electrochemical impedance spectroscopy (EIS) and scanning electron microscope (SEM). Inhibiting mechanism of GO on calcium leaching of the composites was also examined. The obtained results show that the diameter of the semi-circle of the Nyquist curves of leached samples with GO addition decreased less than that of controlled samples. After leaching for 35 days, loss rate of model impedance R_CCP_ of leached samples with 0, 0.05, 0.1, 0.15, and 0.2 wt.% GO addition was 94.85%, 84.07%, 79.66%, 75.34%, and 68.75%, respectively. Therefore, GO addition can significantly mitigate calcium leaching of cement composites, since it can absorb Ca^2+^ ions in cement pore solution, as well as improve the microstructure of the composites. In addition, coupling leaching depth and compressive strength loss were accurately predicted by using the impedance R_CCP_.

## 1. Introduction

Cement composites are the most commonly used man-made materials worldwide and usually exposed to aggressive environments where physical and chemical attacks impair the durability of the composites [1,2,3,4]. Loss of durability further leads to the decrease of the service life of structures, the increase of the environmental burdens, and the increase of the economic costs from maintenance. In particular, calcium leaching is one of the most significant factors that affect the durability of the composites, and generally occurs in infrastructures that have contacted with aqueous environments with the pH value lower than 12.5 over long periods of time such as dams, harbors, cisterns, bridge piers, underground pipes, and nuclear waste storage facilities [5,6].

Calcium leaching is a combined diffusion-dissolution process inside hardened cement composites. Aggressive environments around a structure can result in the concentration gradients of Ca^2+^ ions in pore solution and surrounding environments, which leads to the diffusion of Ca^2+^ ions into surrounding environments [7]. This transfer process modifies the chemical balance of Ca^2+^ ions in cement system [8] and further results in the dissolution of calcium hydroxide (CH), ettringite, and calcium silicate hydrate (C-S-H) [9], which leads to the microstructural changes of the composites. The changes in the microstructure produce detrimental effects on the composites such as the decrease in mechanical properties. According to Gaitero et al. [10], after 28-day curing and 9-day leaching accelerated by 6 mol/L ammonium nitrate (NH_4_NO_3_) solution, plain cement paste with water to cement ratio (w/c) of 0.4 suffered a compressive strength reduction of approximately 75%. Similarly, Arribas et al. [11] found that after 21-day leaching, plain cement paste with w/c of 0.5 suffered the strength reduction of approximately 85%.

To mitigate calcium leaching of cement composites, one effective approach includes the addition of nanomaterials [10,12,13,14]. Among diverse nanomaterials, graphene oxide (GO) is a mono-layer of mixed sp^2^ and sp^3^ hybridized carbon atoms with abundant functional oxygen groups such as carboxyl (–COOH), hydroxyl (–OH), carbonyl (–C=O), and epoxy [15]. As the derivative of graphene, GO shares some characteristics with graphene, such as super-high specific surface (~2600 m^2^/g), high Young’s modulus (~1100 GPa), and tremendous mechanical strength (~1060 GPa) [16]. Due to these properties, GO has been generally used to reinforce the composites at the micro-molecular level [17,18,19]. In terms of calcium leaching, Long et al. [20] investigated the effect of GO on calcium leaching of cement pastes by chemical composition analysis and microstructure analysis. The results indicate that GO addition can decrease CH dissolution, refine the pore structure of leached pastes, and effectively mitigate calcium leaching of the composites. Although the application of GO on leaching inhibition of the composites has been reported, the studies about the inhibiting mechanism of GO on calcium leaching have still been limited.

Currently, a variety of traditional methods have been employed to characterize calcium leaching of cement composites, including phenolphthalein solution test [21], thermo-gravimetric analysis [22], chemical analysis [7], and porosity analysis [23]. However, these methods require samples to be removed from the original structure and further manufactured. Thus, these methods are generally used in laboratory settings, rather than applied in practice engineering, especially in the structure where the timescale exceeds the designated service life such as cross-sea bridges. To monitor the performance changes of infrastructures exposed to calcium leaching, electrochemical impedance spectroscopy (EIS) has been used as an advanced non-destructive testing method to characterize calcium leaching of plain cement pastes [24]. The principle of EIS method is by means of an applied alternate current regime to detect the electrochemical impedance response of cement composites that is affected by aggressive environments [25]. In addition, the relationship between electrochemical impedance and leaching depth has been built. Although EIS method has been used to interpret leaching behaviors of plain cement pastes, the application of EIS method to characterize calcium leaching of GO reinforced cement composites has been still scarce. It should be noted that GO addition makes the electrochemical behaviors of the composites more complicated. According to Long et al. [26], GO addition can significantly increase the electrochemical impedance of the composites.

Based on the aforementioned review, calcium leaching of cement composites reinforced with GO (containing 0, 0.05, 0.1, 0.15, and 0.2 wt.% of cement) was systematically investigated in this study. The experiments were conducted under 6 mol/L ammonium chloride (NH_4_Cl) solution for 7, 14, 21, 28, and 35 days. EIS method was applied as a novel testing technology to characterize calcium leaching of GO/cement composites. Scanning electron microscope (SEM) test was employed to reveal the inhibiting mechanism of GO on calcium leaching of the composites. In addition, macroscopic characterizations including leaching depth and compressive strength were used to explain the feasibility of EIS method on practice engineering. Combined advanced characterizations from the macroscopic, microstructural, and electrochemical perspectives contribute to not only reveal deep insights on leaching behaviors of GO/cement composites, but also monitor the performance changes of leached infrastructure incorporating GO.

## 2. Materials and Methods 

### 2.1. Raw Materials

**Figure 1:**
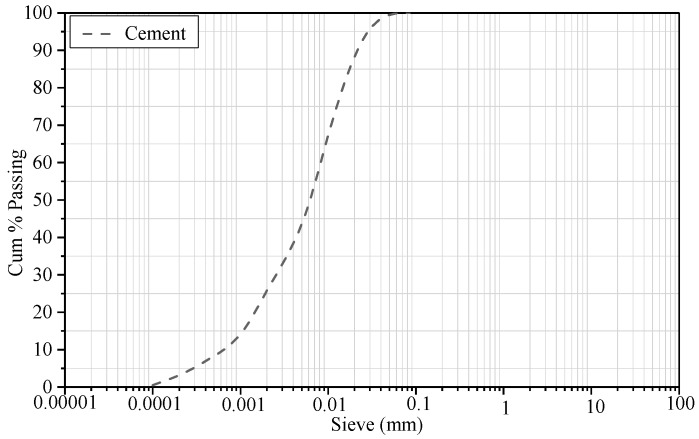
Particle size distribution of OPC.

Ordinary Portland cement (OPC, PI 42.5R) that conformed to the requirements of Chinese Standard GB175 [27] was used in this research. The chemical composition and physical properties of OPC are given in Table 1. The particle-size distribution of OPC was determined using a laser particle-size analyzer, as shown in Figure 1. Graphite oxide powder (Sixth Element Ltd., Changzhou, China) was used for the preparation of the GO solution in the experiment. The properties of graphite oxide powder are given in Table 2.

Polycarboxylate-based high-range water-reducing admixture (PCE) was used to improve the fluidity of the fresh cement composites, conforming to the requirements of Chinese Standard JG/T223 [28]. The maximum rate of water reduction was in the range of 30–35%. In addition, GO will become agglomerate in such a high alkaline environment like cement pore solution due to strong Van der Waal’s force and ionization interference. Therefore, PCE also served as a surfactant to disperse GO in pore solution through electrostatic repulsion and steric stabilization. The characteristics of the chemical admixtures are listed in Table 3.

### 2.2. Test Methods

#### 2.2.1. Preparation and Characterization of GO Solution

A specified amount of graphite oxide powder was mixed with deionized water for 30 min using a magnetic stirrer. The resulting aqueous suspension of graphite oxide with a concentration of 5 g/L was sonicated with an ultrasonic homogenizer (400 W, 25 Hz, model JY98-IIIN, Ningbo Xinzhi Biotechnology Ltd., Ningbo, Zhejiang, China) for 2 h. The ultrasonicator was carried out in cycles—an operation period of 2 s followed by an interval of 2 s—to avoid the suspension overheating. After ultrasonication, the morphology of the obtained GO was scanned by atomic force microscopy (AFM, type ICON-PT-PKG, Bruker, San Diego, CA, USA) and transmission electron microscopy (TEM, type Talos F200X, FEI, Hillsboro, OR, USA). The chemical bonding characteristics of GO were examined by Fourier transform infrared spectroscopy (FTIR, type AVANCE 600 MHz, Bruker, Switzerland). In addition, GO was detected by Raman scattering (type inVia Reflex, Renishaw, London, UK).

#### 2.2.2. Mix Proportioning and Sample Preparation

The mix proportions of GO/cement composites are listed in Table 4. Different amounts of GO used in this study were 0.00%, 0.05%, 0.10%, 0.15%, and 0.20% by weight of cement, respectively. It can be generally accepted that GO is an excellent sorbent due to super-high specified surface area, which results in the absorption of the maximum amount of free water. Thus, the PCE/GO ratio was selected as 3.0 to ensure the adequate workability of the samples.

To achieve the good dispersion of GO in pore solution, GO was preferentially mixed with the same content of PCE in this study [29]. Thus, the mixing procedure of the samples was as followed. First, the GO solution with a specified dosage of PCE (represented as mixture 1) and the water with the rest of PCE (represented as mixture 2) were thoroughly stirred, respectively. Then, cement was added to the mixture 2 and mixed at low rotation speed (62 ± 5 r/min) for 30 s, followed by the addition of the mixture 1 and mixed for 1 min and 30 s. Finally, it was mixed at high rotation speed (125 ± 10 r/min) for another 1 min and 30 s. The obtained GO/cement paste was cast into two molds (dimensions: 30 × 30 × 30 mm^3^ and 40 × 40 × 40 mm^3^) at 20 ± 2 °C and >95% relative humidity, and covered using a plastic film to prevent water loss. After demolding, the samples were cured in a moisture room under the same conditions for 28 days, confirming to the requirements of the Chinese standard GB/T 50081 [30].

#### 2.2.3. Leaching Solution Selection

Calcium leaching in nature is a slow process. It was reported that the leaching front of concrete exposed to still field water for 100 years was only about 5 to 10 mm [31]. Therefore, most of the researches have applied accelerated leaching methods, for example, using 6 mol/L NH_4_NO_3_ solution [32], or 6 mol/L NH_4_Cl solution [33], or deionized water [34], or using electrochemical method [35]. Among these methods, 6 mol/L NH_4_NO_3_ solution has been regarded as the most effective method, which can accelerate the leaching speed two orders and still get the same end products. However, this is also the raw materials for production of explosives. Considering the safety of the experiments, the NH_4_Cl (>99.5% purity, Meilune Biotechnology Ltd., Liaoning, China) was used in this study. The accelerated principle of 6 mol/L NH_4_Cl solution is to promote chemical reaction between CH and NH_4_Cl, which forms gaseous ammonia and calcium chloride that is highly soluble in water (see Equation (1)).

(1) $$\mathrm{Ca}{\left(\mathrm{OH}\right)}_{2}{+2\mathrm{NH}}_{4}{\mathrm{Cl}=\mathrm{Ca}}^{2+}{+2\mathrm{OH}}^{-}{+2H}^{+}{+2\mathrm{NH}}_{3}\left(↑\right){+2\mathrm{Cl}}^{-}{=\mathrm{CaCl}}_{2}{+2\mathrm{NH}}_{3}\left(↑\right){+2H}_{2}O$$

#### 2.2.4. Leaching Test

After curing for 28 days, the samples were placed in the sealed containers filled with 6 mol/L NH_4_Cl solution (solubility: 37.2 g/100 mL at 20 ± 2 °C) to undergo calcium leaching for 7, 14, 21, 28, and 35 days. The volume ratio of an NH_4_Cl solution to a sample was kept constant at 20 to observing phenomena in a short time. In addition, the leaching tests were performed at 20 ± 2 °C and the leachant was not renewed during the tests. After leaching, the samples were removed from the containers, and further manufactured for the next experiments.

#### 2.2.5. Leaching Depth Test

**Figure 2:**
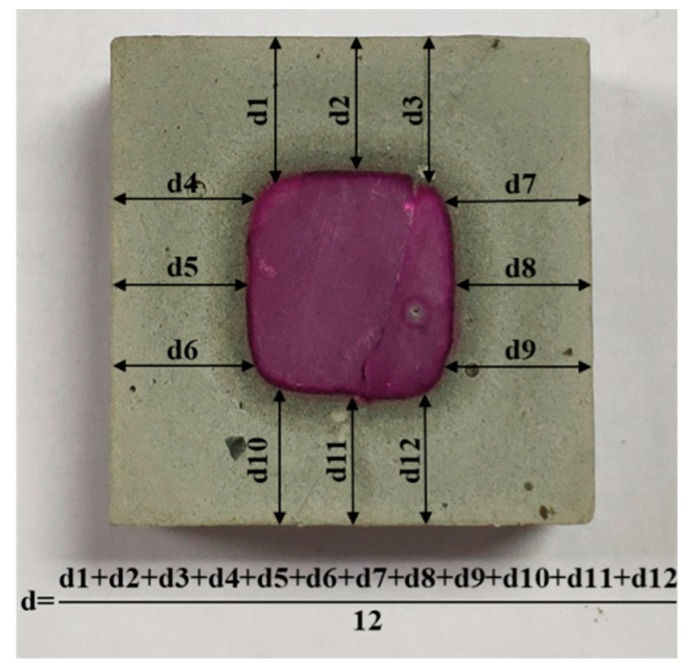
Leaching depth test for the sample exposed to 6 mol/L NH_4_Cl solution.

In this experiment, phenolphthalein pH indicator solution was applied to measure the leaching depth of a leached sample. It was prepared by diluting a mixture of 1 g of phenolphthalein and 90 mL of 95.0 *V*/*V*% ethanol aqueous solution to 100 mL with deionized water. First, a leached sample with a dimension of 30 × 30 × 30 mm^3^ was transversely split into two sections. Then, one of exposed fresh surfaces was immediately cleaned and sprayed with the phenolphthalein solution. Finally, four leaching fronts of the sample were determined using a digital caliper with a measurement accuracy of 0.01 mm. Each front was tested at three appropriate points (Figure 2) and three samples from each group were examined. As a result, the leaching depth of a group was calculated by averaging the thirty-six tested points.

#### 2.2.6. Compressive Strength Test

Three leached samples with a dimension of 40 × 40 × 40 mm^3^ from each group were prepared for the compressive strength test, which was performed at the computerized universal testing machine (YHZ-300) with a loading rate of 2400 N/s, confirming to the Chinese standard GB/T 17671-1999 [36]. Similarly, the strength for each group was calculated by averaging the three tested values.

#### 2.2.7. Electrochemical Impedance Spectroscopy (EIS) Measurement

**Figure 3:**
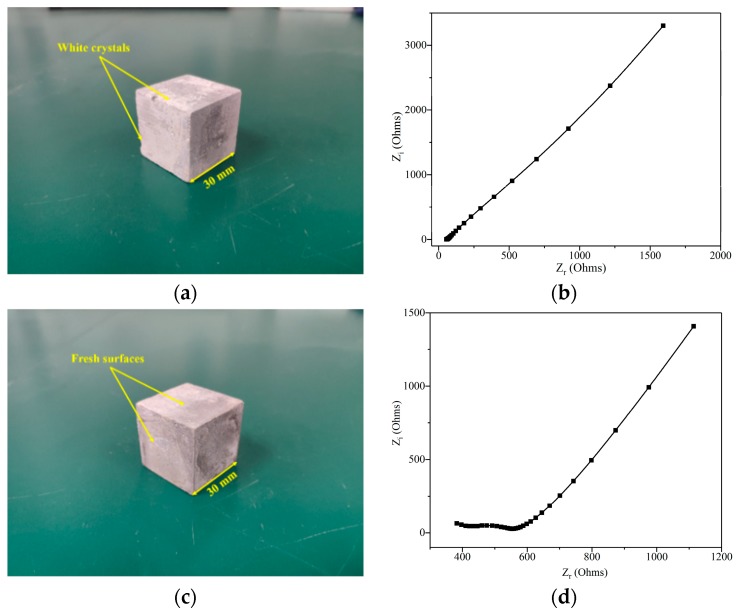
Morphology and the Nyquist curve of G1 after leaching for 7 days: (**a**) morphology of G1 before immersion; (**b**) the Nyquist curve of G1 before immersion; (**c**) morphology of G1 after immersion; and (**d**) the Nyquist curve of G1 after immersion.

To characterize leaching process of the GO/cement composites, a leached sample with a dimension of 30 × 30 × 30 mm^3^ was tested using the electrochemical workstation (type PARSTAT 4000, AMETET Ltd., San Diego, CA, USA) over the frequency range of 1 Hz to 1 MHz at a sinusoidal potential perturbation of 100 mV. It should be noted that leaching in 6 mol/L NH_4_Cl solution can generate unnecessary white crystals on the surface of a leached sample (Figure 3a). The crystals can significantly increase the electrical conductivity of the sample, as shown in the comparison of Figure 3b,d. To be consistent with real conditions on infrastructure, a leached sample was immersed in the tap water (the volume ratio between a sample and the water was 1:3) before test and gently shook to remove the white crystals. A new sample used for this test can be seen in Figure 3c. After the preparation for the samples, the test procedures are as followed. First, the moisture on the surface of the sample need to be wiped. Then, the sample was placed between two parallel electrodes mounted in a tested mold, and directly contacted with the electrodes. Finally, the tested results were analyzed by using the ZSimpWin software.

#### 2.2.8. Microstructural Analysis

To characterize the microstructural changes of GO/cement composites exposed to calcium leaching, the leached samples were detected by scanning electron microscope (SEM) tests. Before test, the leached samples were immersed in anhydrous ethanol to avoid carbonation and to replace cement pore solution for further discussion. After that, the samples were cut from the original one based on a specified region. Then, the sample was placed in an oven at a temperature of 60 °C for 1 d, followed by the SEM tests using a GeminiSEM 300 (Zeiss, Thuringia, Germany). In addition, an energy disperse spectroscopy (EDS) was used to determine the elemental composition of the region of interest.

## 3. Results and Discussion

### 3.1. GO Characterization

**Figure 4:**
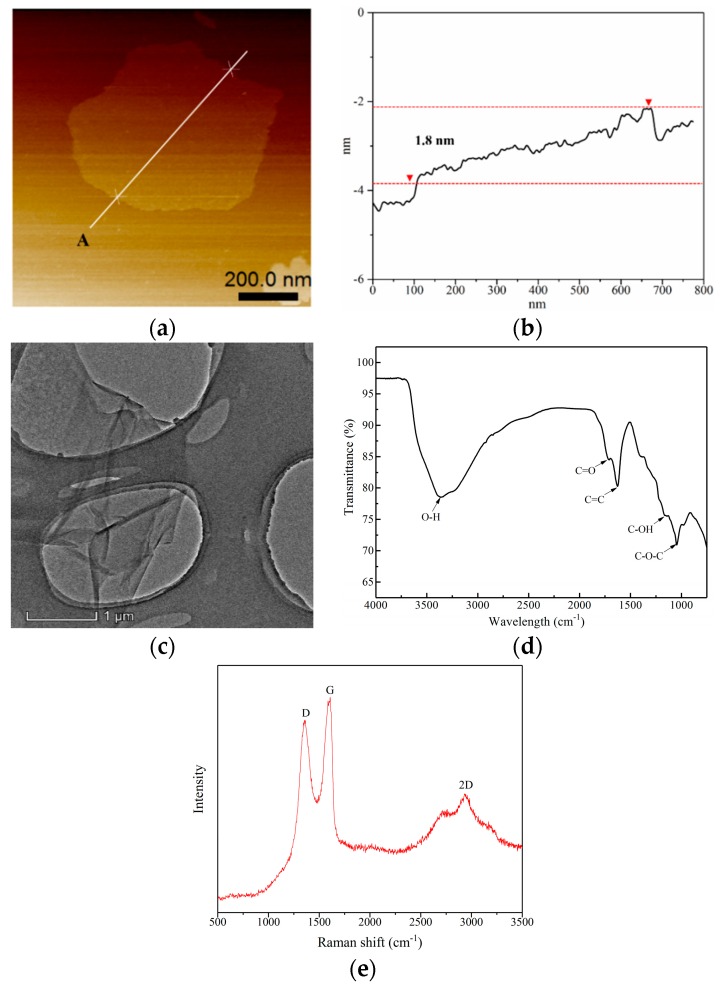
Characterization of GO: (**a**) AFM image; (**b**) AFM spectra; (**c**) TEM image; (**d**) FTIR transmittance spectra; and (**e**) Raman spectrum.

The AFM image and spectra of GO are shown in Figure 4a,b, respectively. These results indicate that GO possessed an irregular plane shape with a dimension of about 0.7 μm and a thickness of about 1.8 nm. Figure 4c shows the TEM image of GO morphology. It can be found that GO was consisted of many wrinkled and folded areas due to intercalating abundant functional oxygen groups. Furthermore, FTIR spectra exhibits the chemical bonds of the functional oxygen groups of GO, as shown in Figure 4d. The typical characteristic peaks of GO were 3400, 1720, 1634, and 1058 cm^−1^, corresponding to –OH, C=O, C=C, and C–O, respectively. These functional oxygen groups made GO easily dispersible in aqueous solutions. In addition, Figure 4e presents the Raman spectrum of GO, where three dominant peaks can be observed. The first band (G-band, at 1620 cm^−1^) was due to the stretching of C-C bond in the graphite mode. The second band (D-band, at 1380 cm^−1^) originated from the diamondoid mode. The third band (2D–band, at 2900 cm^−1^) was the second order of the D peaks. In particular, the I_D_/I_G_ mass ratio of GO is higher than that of graphite, since GO has a disordered structure due to the presence of functional oxygen groups [37].

### 3.2. Macroscopic Characterizations for Leached GO/Cement Composites

#### 3.2.1. Leaching Depth Analysis

Leaching depth tests are carried out by spraying the phenolphthalein solution on the clean surface of the leached samples. The experiments are based on the principle that when sprayed with phenolphthalein, the intact zone of the samples becomes pink due to the existence of alkali, while the degraded zone still keeps grey. Generally, leaching depth can be used to describe the leaching process of cement composites.

**Figure 5:**
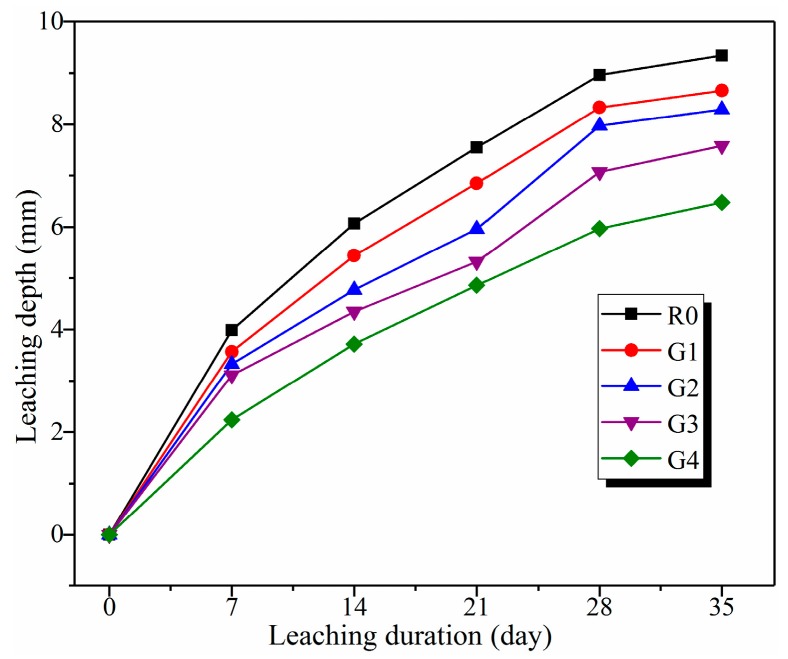
Time-varying leaching depths for the samples exposed to 6 mol/L NH_4_Cl solution.

Figure 5 shows the average of the time-varying leaching depths for the samples exposed to 6 mol/L NH_4_Cl solution. As can be seen from the figure, the growth rate on the leaching depths for all samples decreased progressively due to the reduction of the concentration gradients of Ca^2+^ ions between cement pore solution and the surrounding environments [20]. Furthermore, leaching depths for the samples decreased with the increase of GO content. After leaching for 35 days, the leaching depths for R0, G1, G2, G3, and G4 were 9.34, 8.66, 8.29, 7.58, and 6.48 mm, respectively. In particular, the leaching depth for G4 after leaching for 35 days was still lower than that (7.55 mm) for R0 after leaching for 21 days. According to previous literature [38,39], GO addition can generate a strong barrier effect on the chloride ion transport of cement composites. Therefore, it suggests that GO addition can restrict the diffusion of Ca^2+^ ions and detrimental ions such as NH_4_^+^ and Cl^−^ ions, thus mitigating calcium leaching of cement composites.

**Figure 6:**
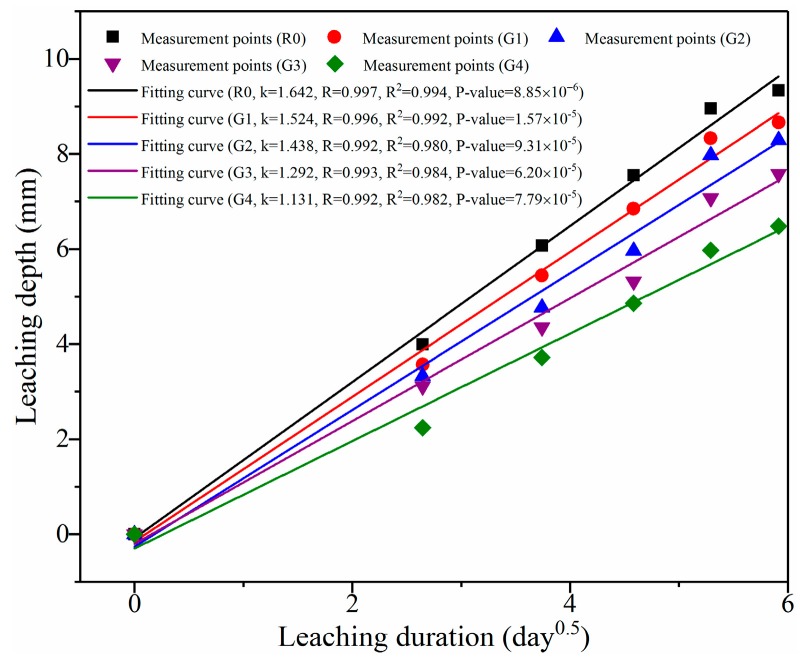
Linear fitting of the leaching depth of the samples in relation to the square root of the leaching duration. Note: the equations of the fitting curves of R0, G1, G2, G3, and G4 are y = 1.642x − 0.083, y = 1.524x − 0.156, y = 1.438x − 0.263, y = 1.292x − 0.204, and y = 1.131x − 0.300.

To further determine the relationship between the leaching depth and the exposure duration, the test data from Figure 5 were fitted to the formula described by Fick’s law: $d=k\sqrt{t}$, where d is the leaching depth (mm), k is the leaching coefficient related to ionic diffusion coefficient, and t is the leaching duration (day). Figure 6 shows the linear fitting on the leaching depth in relation to the square root of the leaching duration. The leaching coefficients (k), correlation coefficient (R), determination coefficients (R^2^), and probability value (*p*-value) are also given in the figure, while the equations of the fitting curves are shown in the figure caption. It can be clearly seen that all R^2^ of the fitting curves were higher than 0.980, indicating that the curves were highly correlated with the measurement points. In this study, the leaching coefficients k of R0, G1, G2, G3, and G4 were 1.642, 1.524, 1.438, 1.292, and 1.131, respectively. According to Forster et al. [6], smaller leaching coefficient (k) indicated slower leaching process. In addition, Tang et al. [40] reported that the leaching coefficient can represent the leaching rate of a sample. Therefore, the leaching rate of R0 was the highest, followed by G1, G2, G3, and G4. In addition, the leaching rate of R0 was above 1.45 times than that of G4. These results further reveal that GO addition can effectively retard leaching process of cement composites.

#### 3.2.2. Compressive Strength Analysis

One of the most important tests from the practical engineering point of view is compressive strength test [10]. As another macroscopic method used in this study, the test provided the parameters that assessed the validity of GO/cement composites for their application in infrastructure exposed to calcium leaching.

**Figure 7:**
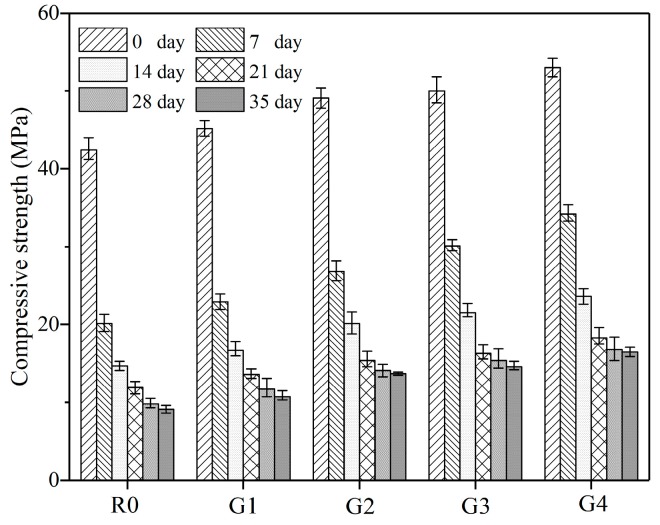
Compressive strengths for the samples leached for 0, 7, 14, 21, 28, and 35 days in 6 mol/L NH_4_Cl solution.

Figure 7 shows the compressive strengths for the samples leached for 0, 7, 14, 21, 28, and 35 days in 6 mol/L NH_4_Cl solution. Before leaching, the compressive strength of the samples increased with the GO content. When compared to R0, the compressive strength of G1, G2, G3, and G4 increased by 6.60%, 15.80%, 17.92%, and 24.98%, respectively. It can be attributed to the super-high specified surface area of GO which acted as nucleation sites to promote cement hydration, and the good dispersion of GO which provided reinforcement at nano-micro structural level [17]. After leaching, compressive strength of the samples decreased gradually with the leaching duration.

**Figure 8:**
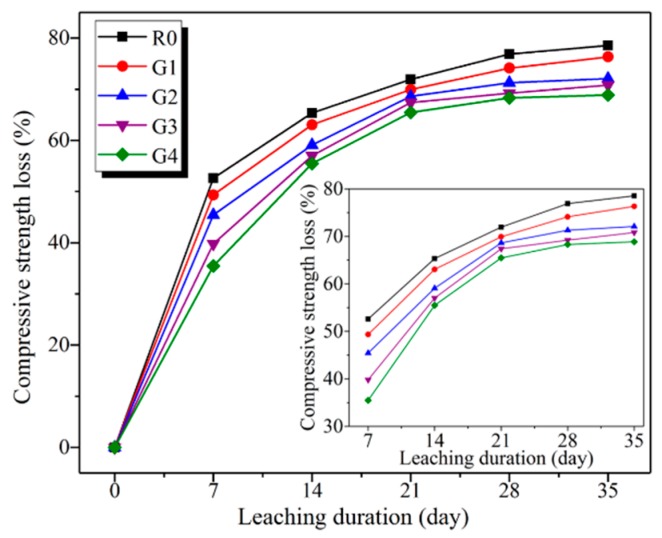
Compressive strength loss of the leached samples in relation to leaching duration.

To reveal the effect of GO on calcium leaching of cement composites, compressive strength loss of the leached samples in relation to leaching duration is shown in Figure 8. It can be clearly seen that the compressive strength loss of the leached samples decreased with the increase of GO content. After leaching for 35 days in 6 mol/L NH_4_Cl solution, the compressive strength loss of R0, G1, G2, G3, and G4 was 78.54%, 76.33%, 72.10%, 70.80%, and 68.87%, respectively. In particular, the compressive strength loss of R0 was above 1.14 times than that of G4. These results indicated that GO addition can significantly mitigate calcium leaching of cement composites. In addition, the loss rate on the compressive strength of the leached samples progressively slowed down with the leaching duration. This phenomenon can be explained by the dissolution of CH and C–S–H. According to previous literature [22,41], the dissolution of CH and C–S–H can be divided into three steps: the quick dissolution of CH, partial dissolution of C–S–H, and quick and total decalcification of the partially leached C–S–H. It suggests that calcium leaching process is firstly dominated by the dissolution of CH, followed by the dissolution of C–S–H. Moreover, the dissolution of CH results in the amount increase of the capillary pores (5 to 5000 nm), while the decalcification of C–S–H results in the amount increase of gel pores (0.5 to 10 nm). When compared to gel pores, the capillary pores are responsible for the reduction in strength of cement composites [5,42]. Consequently, the rapid reduction at early age can be attributed to the additional capillary pores produced by the dissolution of CH, while the deceleration can be due to the additional gel pores formed by the decalcification of C–S–H.

### 3.3. Electrochemical Characterizations for Leached GO/Cement Composites

To characterize calcium leaching of GO reinforced cement composites, the EIS method was applied as an advanced technique in this study. First, three modifications of the equivalent circuit model were proposed. Then, the effects of leaching duration and GO addition on the Nyquist curves of the leached samples were discussed. Finally, the model impedance R_CCP_ were used to further present the inhibiting effect of GO on calcium leaching of the composites, and predict coupling leaching depth and compressive strength loss of the leached samples.

#### 3.3.1. Modifications of Equivalent Circuit Model for Leached Samples

**Figure 9:**
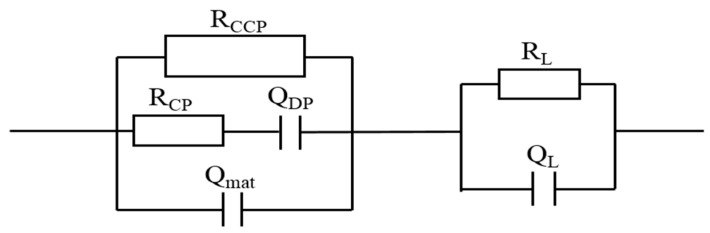
Equivalent circuit model for calcium leaching of cement composites, where R_CCP_ is the resistance of the CCPs in the intact zone; Q_DP_ is the double layer capacitance of the DCPs in the intact zone; R_CP_ is the resistance of the DCPs in the intact zone; Q_mat_ is the double layer capacitance of the ICPs in the intact zone; R_L_ is the resistance of the CCPs in the degraded zone; and Q_L_ is the double layer capacitance of the ICPs in the degraded zone.

According to Song et al. [24], a novel equivalent circuit model used to explain calcium leaching behaviors of plain cement pastes was proposed, as shown in Figure 9. However, there are several confusions in this model. Thus, this model is updated here through three modifications. First, cement composites are an electrochemical system which has three current channels: continuous conductive paths (CCPs) composed of continuously connected micro-pores, discontinuous conductive paths (DCPs) consisted of discontinuous micro-pores, and “insulator” conductive paths (ICPs) from continuous cement matrix [43]. In the model, the CCPs, ICPs, and DCPs should be viewed as a resistor, a capacitor, and a series connection of resistor and capacitor, respectively. Then, there are two regions in the leached samples: the degraded zone and the intact zone. In the degraded zone, the DCPs can be neglected rather than the ICPs since the DCPs are degraded in such a greater extent. Finally, the description code of this model can be described as (Q_mat_ (Q_DP_ R_CP_) R_CCP_) (Q_L_ R_L_), and the modified equation of the total impedance Z of the model (Q_mat_ (Q_DP_ R_CP_) R_CCP_) (Q_L_ R_L_) can be expressed by Equation (2):

(2) $$Z=\frac{R_{\mathrm{CCP}}{(1+R}_{\mathrm{CP}}{j\omega Q}_{\mathrm{DP}})}{\left({1+R}_{\mathrm{CCP}}{j\omega Q}_{\mathrm{mat}}\right)\left({1+R}_{\mathrm{CP}}{j\omega Q}_{\mathrm{DP}}\right){+R}_{\mathrm{CCP}}{j\omega Q}_{\mathrm{DP}}}+\frac{R_{L}}{{1+R}_{L}{j\omega Q}_{L}}$$

where $j=\sqrt{-1}$ and ω is the angle frequency. The physical significance of each circuit elements are captioned in Figure 9. Based on a novel model proposed by Song et al. [24], this modified one was applied in this study for analyzing calcium leaching of GO reinforced cement composites.

#### 3.3.2. Effect of Leaching Duration on the Nyquist Curve of Leached Samples

**Figure 10:**
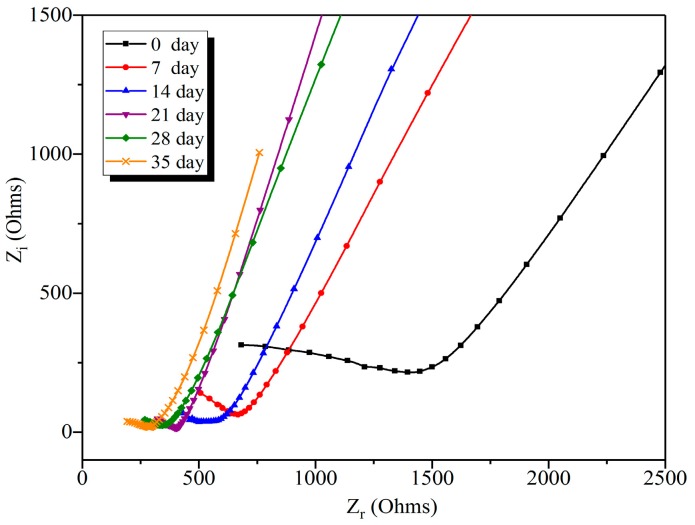
The Nyquist curves of G2 samples undergoing different leaching durations.

Figure 10 shows the Nyquist curves of G2 samples exposed to different leaching durations. It can be found that the diameters of the semi-circles of the Nyquist curves decreased with the increase of leaching duration. According to Long et al. [26], the diameters of the semi-circle of the Nyquist curves are proportional to the density degree of pore structure of the composites. Thus, the decrease on the diameters of the semi-circles can be attributed to the increment of the porosity of leached composites from the dissolution of ettringite and CH, and the decalcification of C-S-H. In particular, there was a sudden reduction on the diameter of the semi-circle of the Nyquist curve for G2 samples after leaching for 7 days, when compared to before leaching. Combined with the results from macroscopic characterizations, this phenomenon can be explained by the evolution of concentration gradients of Ca^2+^ ions, and the appearance of additional capillary pores at early age. In addition, same trends can be found in the Nyquist curves of R0, G1, G3, and G4, as shown in Figure S1.

#### 3.3.3. Effect of GO Addition on the Nyquist Curve of Leached Samples

**Figure 11:**
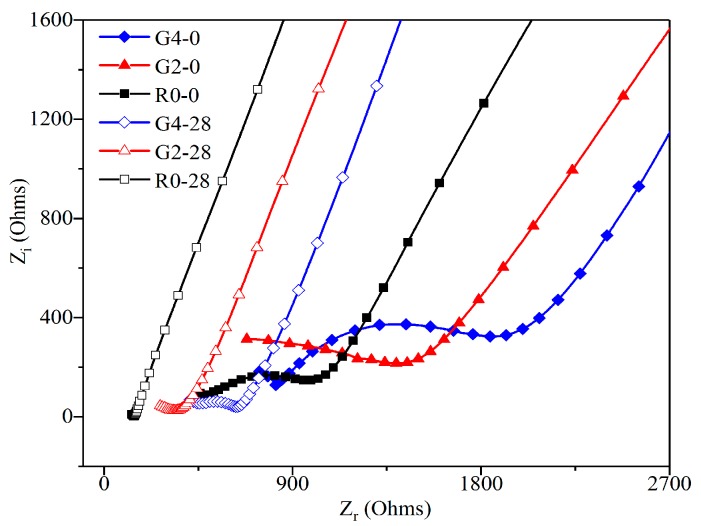
The Nyquist curves of R0, G2, and G4 before leaching and after leaching for 28 days.

To investigate the effect of GO addition on calcium leaching of cement composites, the Nyquist curves of R0, G2, and G4 before and after leaching for 28 days are shown in Figure 11. As can be seen from the figure, the diameters of the semi-circle of the Nyquist curves of the samples decreased after leaching for 28 days, when compared to before leaching. Furthermore, the diameters of the semi-circle of the Nyquist curves of the leached samples with GO addition decreased less than that of the samples without GO addition. This phenomenon became more obvious in the case of the higher GO content. These results show that GO addition can effectively inhibit the impedance loss of leached composites. According to previous literature [16,44,45], GO can be agglomerate in Ca(OH)_2_ solution due to the reaction between the functional oxygen groups with Ca^2+^ ions. It suggests that GO addition can absorb Ca^2+^ ions in cement pore solution via its functional oxygen groups, therefore mitigating calcium leaching of cement composites. In addition, the diameters of the semi-circle of the Nyquist curve of the unleached samples increased with the GO contents. This is due to the fact that GO addition promoted the cement hydration, therefore forming a denser microstructure. More information about the Nyquist curves of the samples after leaching for 0, 7, 14, 21, 28, and 35 days is shown in Figure S2.

#### 3.3.4. Model Impedance R_CCP_ Analysis and its Fitting Results

The values of the model impedance R_CCP_ from the equivalent circuit model (Q_mat_ (Q_DP_ R_CP_) R_CCP_) (Q_L_ R_L_) at different leaching durations are listed in Table 5. It can be clearly seen that the value of R_CCP_ decreased with increase of leaching duration. Moreover, the loss percentages of R_CCP_ in R0, G1, G2, G3, and G4 were 94.85%, 84.07%, 79.66%, 75.34%, and 68.75%, respectively. In particular, the loss percentage of R_CCP_ in R0 was almost 1.4 times than that in G4. These results quantitatively indicate that GO addition can inhibit calcium leaching of cement composites.

**Figure 12:**
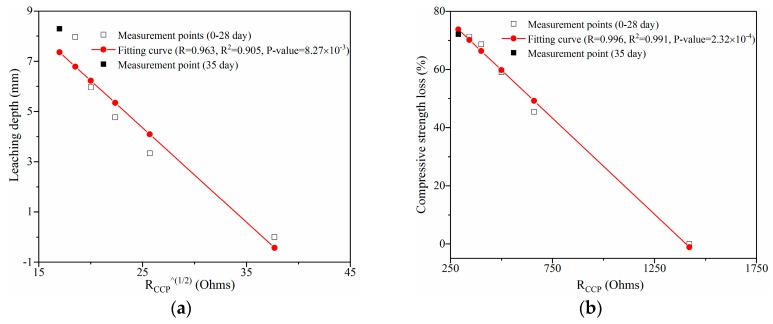
Prediction on leaching depth and compressive strength loss of G2 using R_CCP_: (**a**) leaching depth and (**b**) compressive strength loss. Note: the equations of the fitting curves of (**a**) and (**b**) are y = 13.750 − 0.376x and y = 92.841 − 0.066x, respectively.

In theory, the impedance values of the leached GO/cement composites can be used not only to reveal the inhibiting effect of GO on calcium leaching, but also to reflect the performance changes of the composites. Figure 12a,b show the predictions of leaching depth and compressive strength loss of G2 during leaching for 28 days, respectively. Predictions of leaching depth and compressive strength loss of R0, G1, G3, and G4 are given in Figure S3. The correlation coefficient (R), determination coefficients (R^2^), and probability value (*p*-value) of the fitting curves are provided in the figures, while the equations of the fitting curves are shown in the figure caption. It can be clearly seen from R^2^ that the curves were highly correlated with the measurement points. In addition, the prediction of compressive strength loss is more accurate than that of leaching depth. Table 6 and Table 7 summarize the measured results and the predicted results of leaching depth and compressive strength loss in all samples at leaching for 35 days. In particular, the maximum variation was only 12.80%. Therefore, the application of EIS method for monitoring the leached infrastructure with GO addition is reliable.

### 3.4. Microstructural Characterization for Leached GO/Cement Composites

**Figure 13:**
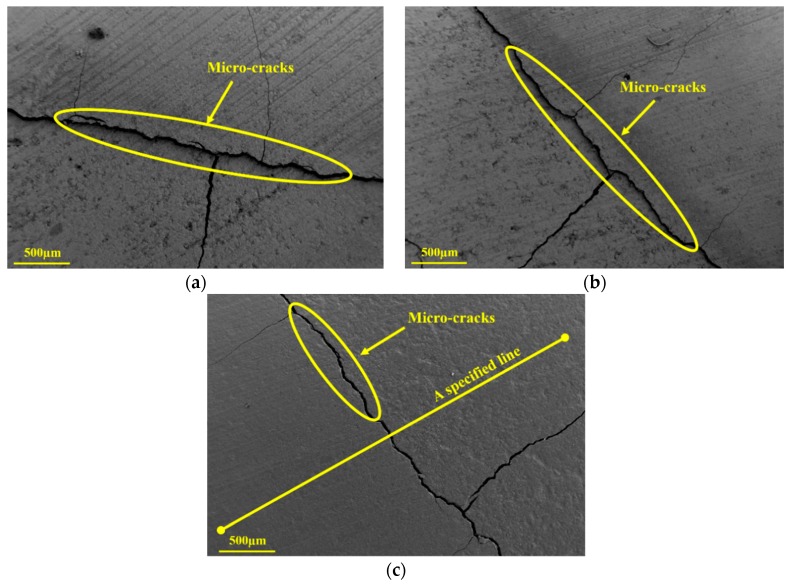
Cutting surfaces of R0, G2, and G4 after leaching for 14 days: (**a**) R0; (**b**) G2; and (**c**) G4.

**Figure 14:**
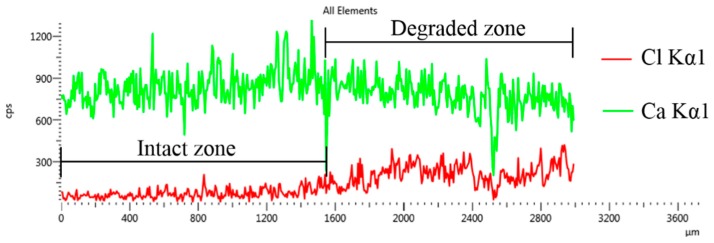
The EDS data for a specified line in Figure 13c.

Durability of cement composites is closely related to their microstructural characteristics. Figure 13a–c show the cutting surfaces of R0, G2, and G4 after leaching for 14 days, respectively. Figure 14 shows the EDS data for a specified line in G4 surface. It can be clearly seen from these figures that there were two different regions in the leached samples, which correspond to the degraded zone and the intact zone in the equivalent circuit model, respectively. With the increase of GO content, the differences between these zones in the leached samples became indistinct. Furthermore, several micro-cracks can be found in the interface between these zones. It suggests that the bonding between the degraded zone and the intact zone was corroded by calcium leaching, and the rapid evaporation of anhydrous ethanol aggravated the separation of these zones. In addition, the micro-cracks in the leached samples were improved by the GO addition. These results exhibit the reinforced effect of GO on microstructure of leached samples.

**Figure 15:**
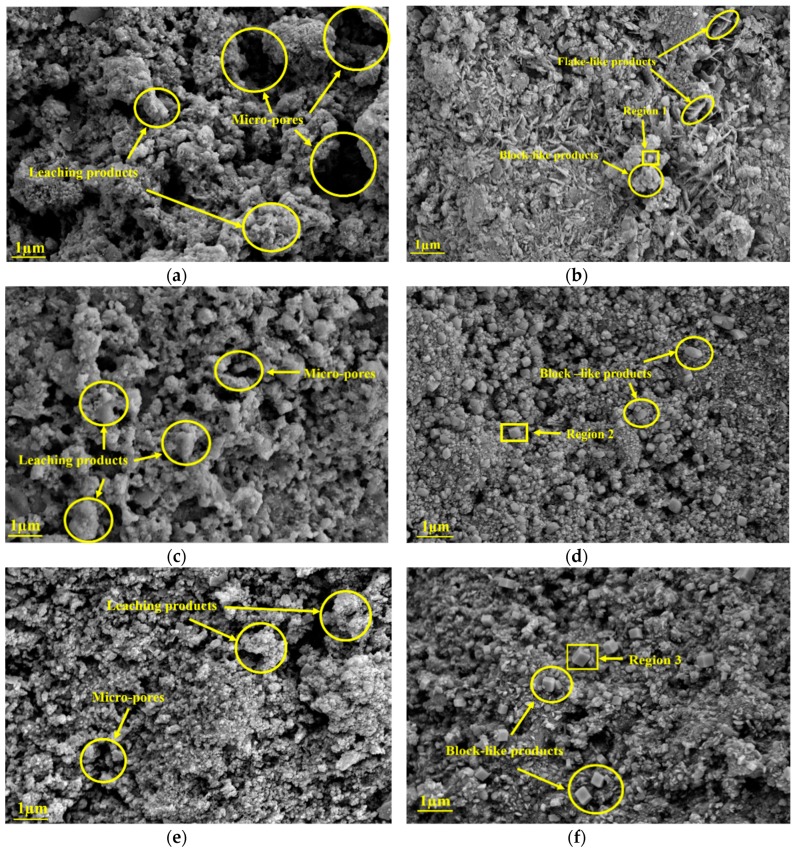
Microstructure characterizations of R0, G2, and G4 after leaching for 14 days: (**a**) the degraded zone of R0; (**b**) the intact zone of R0; (**c**) the degraded zone of G2; (**d**) the intact zone of G2; (**e**) the degraded zone of G4; and (**f**) the intact zone of G4.

The degraded zone and intact zone of R0, G2, and G4 after leaching for 14 days were characterized, as shown in Figure 15. From the comparison of Figure 15a,c,e, the degradation of the microstructure for R0 suffered the most, including the appearance of many leaching products and micro-pores, followed by G2 and G4. This indicates that GO addition can refine the microstructure of leached samples. From the comparison of Figure 15b,d,f, the morphology of portlandite crystals in the intact zone of the leached samples transforms from the hexagonal-like form into the block-like form. Besides, the amount and volume of block-like products increase with the GO content. These phenomena can be attributed to the promoted effect of GO on cement hydration.

It should be noted that Cl^−^ ions cannot be found in the block-like hydrated products of R0, as shown in the region 1 of Table 8. Since GO addition increases the resistance of the sample towards ionic transport [26], Cl^−^ ions should be also absent in G2 and G4 under the same experimental conditions. However, the regions 2 and 3 of Table 8 demonstrate the existence of Cl^−^ ions in the G2 and G4, respectively. This indicates that GO surface possessed positive charges due to the accumulation of Ca^2+^ ions, which leads to the attraction of Cl^−^ ions. It should be noted that the effects of K^+^ and Na^+^ ions on surface potential of GO were not considered, since these ions cannot react with the functional oxygen groups of GO [46].

### 3.5. Inhibiting Mechanism of GO on Calcium Leaching of Cement Composites

Through a series of advanced characterizations, it can be concluded that GO addition can effectively mitigate calcium leaching of cement composites. From SEM and EDS characterizations, this inhibition can be partially due to the functional oxygen groups of GO that can absorb Ca^2+^ ions in cement pore solution, and partially owing to the reinforced effect of GO on the microstructure of cement composites. Based on this mechanism, the systematical explanations for calcium leaching of GO reinforced cement composites were as followed.

*Electrochemical impedance of unleached samples increased with GO content*—On one hand, GO addition can promote cement hydration [47], which can densify the microstructure of the composites, therefore increasing the resistance towards the migration of conductive ions. On the other hand, promoted cement hydration due to GO addition can also increase ion contents in pore solution, which resulted in the increment on the conductivity of the solution. According to Dong et al. [25], the impedance was inversely proportional to the amounts of conductive ions and ionic paths. Therefore, the number of ionic paths was dominated in electrochemical impedance of the composites, when compared to the number of conductive ions. In addition, there was a non-negligible charge shielding effect [48] that separation of every proton from GO surface in the electrolyte was resisted by the electrical field created by “free” ions in pore solution. Therefore, massive metallic ions, including K^+^, Na^+^, and Ca^2+^ ions, can be gathered by GO surface, which hindered the increment of the conductivity of the solution. It should be noted that since the conductivity of pore solution mainly derived from K^+^, Na^+^, and OH^−^ ions [49], the decrement on Ca^2+^ ions in pore solution at this stage contributed little to the increment on the conductivity of the solution.

*Loss rate of electrochemical impedance of leached samples decreased with the increase of GO content*—In general, calcium leaching is a combined diffusion and dissolution process inside hardened cement composites, which indicates that leaching can decrease the number of conductive ions and the conductivity of pore solution. However, the changes on the conductivity of the solution become more complicated, when the composites leached in 6 mol/L NH_4_Cl solution. This is due to the fact that K^+^, Ca^2+^, Na^+^, and OH^−^ ions in pore solution can diffuse along with the concentration gradients. In addition, NH_4_^+^ and Cl^−^ ions in aggressive environments can intrude on. Since it is very difficult to physically extract cement pore solution for measuring its conductivity [50,51] and the amount of ionic paths plays a dominated role on electrochemical impedance, the amount of ionic paths is used in this study to explain this deceleration phenomenon. On one hand, since GO can absorb Ca^2+^ ions in pore solution, the reduction of Ca^2+^ ions in pore solution can decrease the dissolution of CH and C–S–H, therefore inhibiting the formation of additional capillary and gel pores in leached samples. On the other hand, GO addition can increase the crack tortuosity of unleached samples [52], which prolongs the escaping distance of Ca^2+^ ions and further decreases the loss of Ca^2+^ ions in pore solution.

## 4. Conclusions

In this study, cement composites reinforced with GO (containing 0, 0.05, 0.1, 0.15, and 0.2 wt.% of cement) were exposed to 6 mol/L NH_4_Cl solution for 7, 14, 21, 28, and 35 days to undergo calcium leaching. EIS method was applied to characterize leaching process of GO/cement composites. SEM and EDS tests were employed to reveal the inhibiting mechanism of GO on calcium leaching of the composites. In addition, macroscopic characterizations including leaching depth and compressive strength were used to explain the feasibility of applying EIS method on practice engineering. Based on the test results, the following conclusions can be drawn:

1. Leaching depth of the samples decreased with the increase of GO content. After leaching for 35 days, leaching depths of R0, G1, G2, G3, and G4 were 9.34, 8.66, 8.29, 7.58, and 6.48 mm, respectively. Furthermore, the leaching coefficient of R0 was above 1.45 times than that of G4. These results demonstrate that GO addition can effectively mitigate calcium leaching of cement composites.
2. After leaching for 35 days, the compressive strength loss of R0, G1, G2, G3, and G4 was 78.54%, 76.33%, 72.10%, 70.80%, and 68.87%, respectively. In particular, the rapid loss of compressive strength at early age was attributed to the additional capillary pores produced by the dissolution of calcium hydroxide.
3. A modified circuit model was proposed to explain the impedance response of the samples exposed to calcium leaching. After leaching for 35 days, loss rate of model impedance R_CCP_ of leached samples with 0, 0.05, 0.1, 0.15, and 0.2 wt.% GO addition was 94.85%, 84.07%, 79.66%, 75.34%, and 68.75%, respectively. In addition, coupling leaching depth and compressive strength loss were accurately predicted by the model parameter R_CCP_, which indicated that EIS method can be applied to monitor the performance change of the leached infrastructure incorporating GO.
4. The leached samples can be divided into two regions: the degraded zone and the intact zone, which is consistent with the model. After leaching for 14 days, the number of leaching products and micro-pores in the degraded zone of the samples decreased with the increase of GO content, indicating that GO addition can refine the microstructure of leached cement composites.
5. GO addition can significantly mitigate calcium leaching of cement composites. This is due to the fact that GO can absorb Ca^2+^ ions in cement pore solution, as well as improve the microstructure of GO/cement composites.

## Figures and Tables

**Table 1:** Chemical composition and physical properties of OPC.

**Chemical Composition**	**Ingredient**	**CaO**	**SiO_2_**	**Al_2_O_3_**	**Fe_2_O_3_**		**MgO**		**SO_3_**		**K_2_O**		**Na_2_O**		**LOI**
	Content (mass %)	64.42	20.52	5.62	3.78		2.11		2.10		0.28		0.20		0.87
**Physical properties**	**Specific surface area (m^2^/g)**		**ρ_0_ (g/cm^3^)**	**Setting time (min)**				**Flexural strength (MPa)**				**Compressive strength (MPa)**			
	0.581		3.00	Initial		Final		3 d		28 d		3 d		28 d	
				112		145		6.50		9.20		34.80		58.00	

**Table 2:** Properties of graphite oxide.

Appearance	Solid Content (mass %)	pH	Viscosity (Pa·s)	Absorbance Ratio A230/A600	Carbon (mass %)	Molar Ratio (O/C)
Brown paste	43 ± 1	≥1.2	≥2000	≥45	47 ± 5	0.6 ± 1

**Table 3:** Characteristics of chemical admixtures.

Designation	State	Density (g/m^3^)	pH	Solid Content (mass %)
RCM–3	liquid	1.102	5.0	49.98
CP–WRM50	liquid	1.114	4.5	50.79

**Table 4:** Mix proportions of GO/cement composites.

Sample	Cement (g)	Water (g)	W/C Ratio	GO (g)	PCE/GO
R0	100	40	0.4	0	–
G1	100	40	0.4	0.05	3.0
G2	100	40	0.4	0.1	3.0
G3	100	40	0.4	0.15	3.0
G4	100	40	0.4	0.2	3.0

Note: the samples are represented according to the contents of GO.

**Table 5:** Fitting results of R_CCP_ based on the modified model (Q_mat_ (Q_DP_ R_CP_) R_CCP_) (Q_L_ R_L_) for the leached samples at different leaching durations (Ohms).

Leaching Duration (day)	R0	G1	G2	G3	G4
0	970	1287	1421	1545	1843
7	320	556	660	801	1012
14	255	421	500	625	883
21	182	289	401	481	710
28	143	255	343	440	635
35	50	205	289	381	576

**Table 6:** Comparisons of measured results and predicted results for leaching depth.

Leaching Duration	Measured vs. Calculated Depth	GO Content				
		0%	0.05%	0.1%	0.15%	0.2%
35 days	Measured depth (mm)	9.34	8.66	8.29	7.58	6.48
	Predicted depth (mm)	10.19	8.10	7.36	6.61	5.82
	Variation (%)	9.10	6.47	11.22	12.80	10.19

**Table 7:** Comparisons of measured results and predicted results for compressive strength loss (%).

Leaching Duration	Measured vs. Calculated Loss	GO Content				
		0%	0.05%	0.1%	0.15%	0.2%
35 days	Measured loss	78.54	76.33	72.10	70.80	68.87
	Predicted loss	82.90	76.70	73.74	71.71	71.25
	Variation	5.55	0.48	2.22	1.29	3.46

**Table 8:** The EDS data for the specified regions in Figure 15: (a) region 1 in Figure 15b; (b) region 2 in Figure 15d; and (c) region 3 in Figure 15f.

Elements	Region 1		Region 2		Region 3	
	wt.%	σ	wt.%	σ	wt.%	σ
Ca	54.1	0.3	46.5	0.2	47.7	0.3
O	33.9	0.3	38.4	0.2	32.0	0.2
C	7.3	0.3	10.4	0.2	10.0	0.3
Si	4.7	0.1	3.7	0.1	7.7	0.1
Cl	–	–	1.0	0.1	2.6	0.1

## Supplementary Materials

The following are available online at http://www.mdpi.com/2079-4991/9/2/288/s1. Figure S1: The Nyquist curves of the leached samples undergoing different leaching durations: (a) R0, (b) G1, (c) G3, and (d) G4; Figure S2: The Nyquist curves of the samples before leaching and after leaching for 7, 14, 21, 28, and 35 days: (a) before leaching, (b) after leaching for 7 days, (c) after leaching for 14 days, (d) after leaching for 21 days, (e) after leaching for 28 days, and (f) after leaching for 35 days; Figure S3: Predictions on leaching depth and compressive strength loss of leached samples using R_CCP_: (a) leaching depth of R0, (b) compressive strength loss of R0, (c) leaching depth of G1, (d) compressive strength loss of G1, (e) leaching depth of G3, (f) compressive strength loss of G3, (g) leaching depth of G4, and (h) compressive strength loss of G4.
