# Supplementary material for: Electrochemical Characterization and Inhibiting Mechanism on Calcium Leaching of Graphene Oxide Reinforced Cement Composites
Source: Nanomaterials (Basel). 2019 Feb 19;9(2):288. doi: 10.3390/nano9020288 (PMC6409634; doi:10.3390/nano9020288)
Supplement: Supplementary file 1 [file nanomaterials-09-00288-s001.pdf]

Supplementary materials:

Electrochemical characterization and inhibiting mechanism on calcium leaching of graphene oxide reinforced cement composites

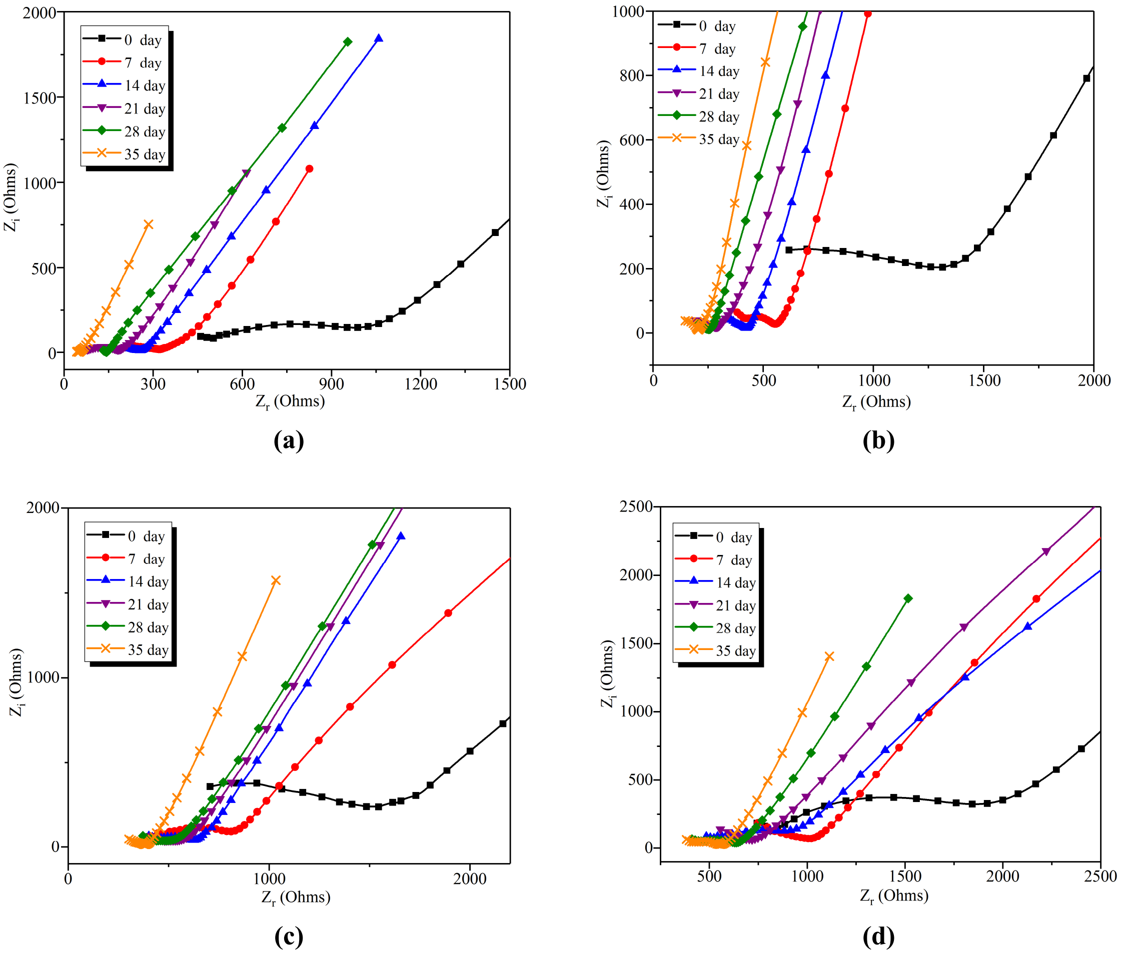

Figure S1. The Nyquist curves of the leached samples undergoing different leaching durations: (a) R0; (b) G1; (c) G3; and (d) G4.

As shown in Figure S1, the diameters of the semi-circles of the Nyquist curves of R0, G1, G3, and G4 decreased with the increase of leaching duration. It can be attributed to the increment of the porosity of leached composites from the dissolution of ettringite and CH, and the decalcification of C-S-H.

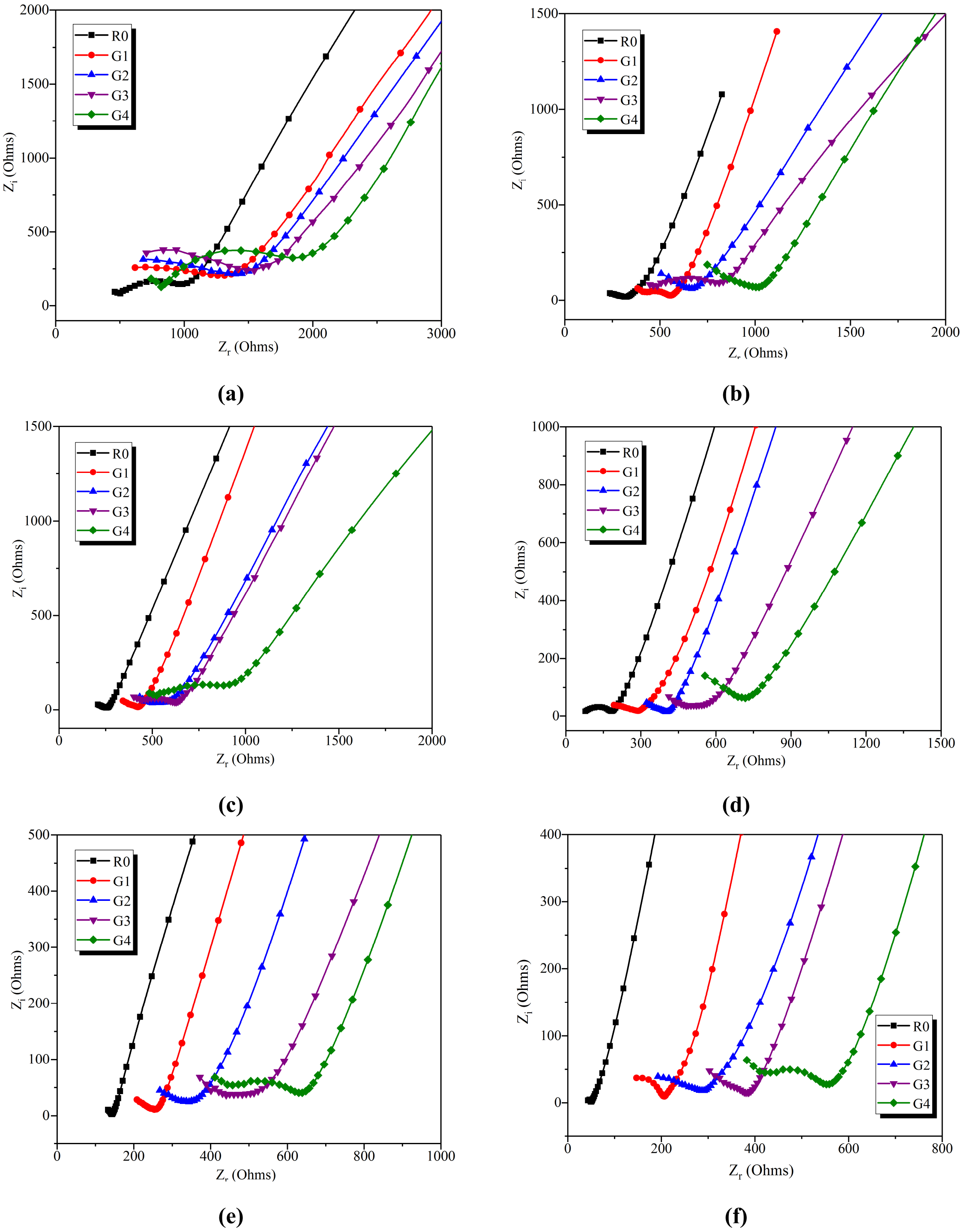

Figure S2. The Nyquist curves of the samples before leaching and after leaching for 7, 14, 21, 28, and 35 days: (a) before leaching; (b) after leaching for 7 days; (c) after leaching for 14 days; (d) after leaching for 21 days; (e) after leaching for 28 days; and (f) after leaching for 35 days.

As shown in Figure S2, the diameters of the semi-circles of the Nyquist curves of the samples after leaching for different durations increased with the GO content.

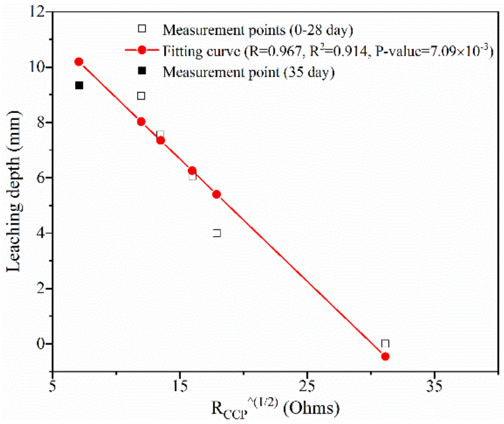
Measurement points (0-28 day)
Fitting curve (R=0.967, R²=0.914, P-value=7.09×10⁻³)
Measurement point (35 day)
Leaching depth (mm)
R_CCP^(1/2) (Ohms)

(a)

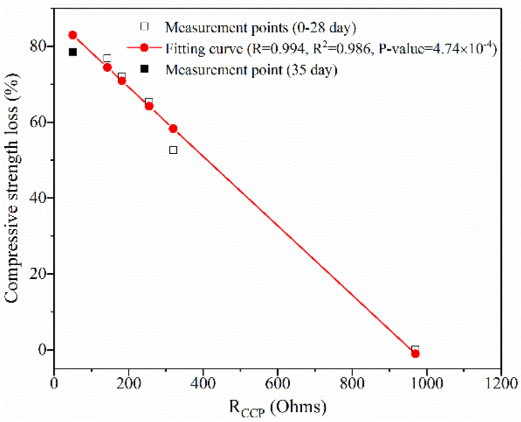
Measurement points (0-28 day)
Fitting curve (R=0.994, R²=0.986, P-value=4.74×10⁻⁴)
Measurement point (35 day)
Compressive strength loss (%)
R_CCP (Ohms)

(b)

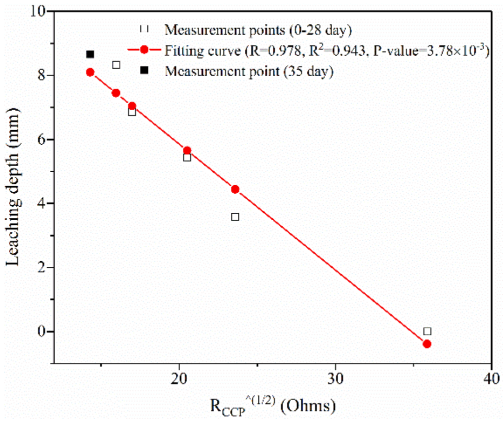
Measurement points (0-28 day)
Fitting curve (R=0.978, R²=0.943, P-value=3.78×10⁻³)
Measurement point (35 day)
Leaching depth (mm)
R_CCP^(1/2) (Ohms)

(c)

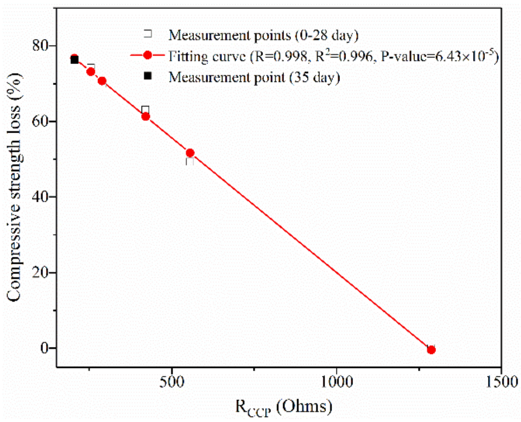
Measurement points (0-28 day)
Fitting curve (R=0.998, R²=0.996, P-value=6.43×10⁻⁵)
Measurement point (35 day)
Compressive strength loss (%)
R_CCP (Ohms)

(d)

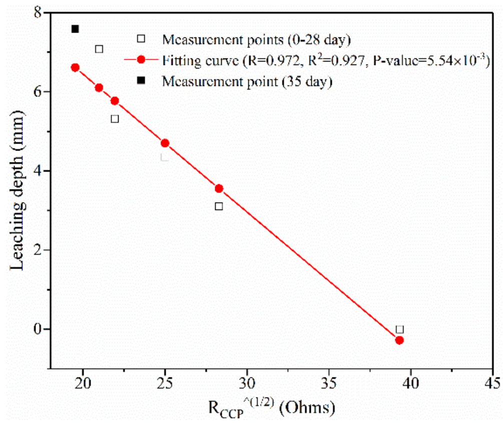
Measurement points (0-28 day)
Fitting curve (R=0.972, R²=0.927, P-value=5.54×10⁻³)
Measurement point (35 day)
Leaching depth (mm)
R_CCP^(1/2) (Ohms)

(e)

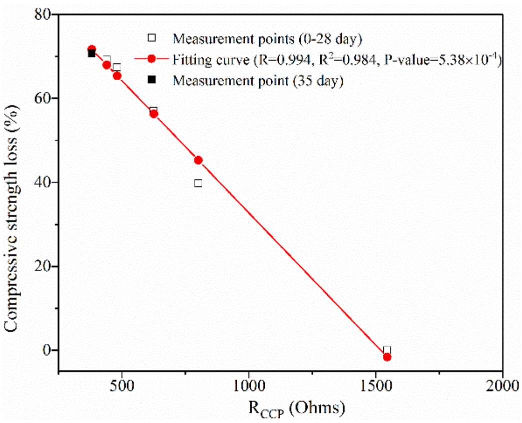
Measurement points (0-28 day)
Fitting curve (R=0.994, R²=0.984, P-value=5.38×10⁻⁴)
Measurement point (35 day)
Compressive strength loss (%)
R_CCP (Ohms)

(f)

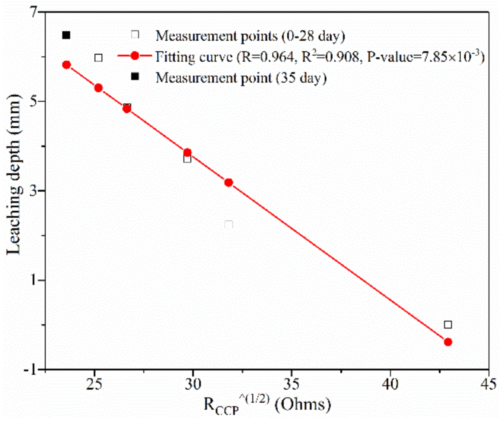

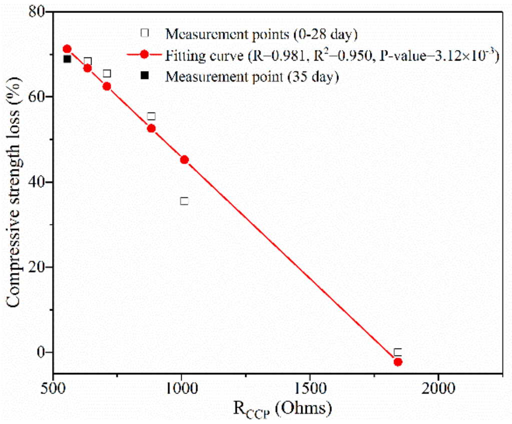

(g) **(h)**

Figure S3. Predictions on leaching depth and compressive strength loss of leached samples using R_{CCP}: (a) leaching depth of R0; (b) compressive strength loss of R0; (c) leaching depth of G1; (d) compressive strength loss of G1; (e) leaching depth of G3; (f) compressive strength loss of G3; (g) leaching depth of G4; and (h) compressive strength loss of G4. Note: the equations of the fitting curves of (a), (b), (c), (d), (e), (f), (g), and (h) are y=13.316–0.442x, y=87.462–0.091x, y=13.738–0.394x, y=91.319–0.071x, y=13.402–0.348x, y=95.702–0.063x, y=13.382–0.320x, and y=103.006–0.057x, respectively.

As shown in Figure S3, the curves were highly correlated with the measurement points. Furthermore, the prediction of compressive strength loss is more accurate than that of leaching depth.
